# Emergency Medicine Training Programs in Low- and Middle-Income Countries: A Systematic Review

**DOI:** 10.5334/aogh.2681

**Published:** 2020-06-16

**Authors:** Megan M. Rybarczyk, Nicholas Ludmer, Morgan C. Broccoli, Sean M. Kivlehan, Michelle Niescierenko, Mark Bisanzo, Keegan A. Checkett, Shada A. Rouhani, Andrea G. Tenner, Heike Geduld, Teri Reynolds

**Affiliations:** 1 Department of Emergency Medicine, Brigham and Women’s Hospital, Harvard Medical School, US; 2 Section of Emergency Medicine, Department of Medicine, University of Chicago Pritzker School of Medicine, US; 3 Department of Emergency Medicine, Boston Medical Center, US; 4 Division of Emergency Medicine, Boston Children’s Hospital, Harvard Medical School, US; 5 Division of Emergency Medicine, Department of Surgery, University of Vermont, US; 6 Department of Emergency Medicine, University of California, San Francisco, US; 7 University of Cape Town/Stellenbosch University, College of Emergency Medicine of South Africa, ZA; 8 World Health Organization, CH

## Abstract

**Background:**

Despite the growing interest in the development of emergency care systems and emergency medicine (EM) as a specialty globally, there still exists a significant gap between the need for and the provision of emergency care by specialty trained providers. Many efforts to date to expand the practice of EM have focused on programs developed through partnerships between higher- and lower-resource settings.

**Objective:**

To systematically review the literature to evaluate the composition of EM training programs in low- and middle-income countries (LMICs) developed through partnerships.

**Methods:**

An electronic search was conducted using four databases for manuscripts on EM training programs – defined as structured education and/or training in the methods, procedures, and techniques of acute or emergency care – developed through partnerships. The search produced 7702 results. Using *a priori* inclusion and exclusion criteria, 94 manuscripts were included. After scoring these manuscripts, a more in-depth examination of 26 of the high-scoring manuscripts was conducted.

**Findings:**

Fifteen highlight programs with a focus on specific EM content (i.e. ultrasound) and 11 cover EM programs with broader scopes. All outline programs with diverse curricula and varied educational and evaluative methods spanning from short courses to full residency programs, and they target learners from medical students and nurses to mid-level providers and physicians. Challenges of EM program development through partnerships include local adaptation of international materials; addressing the local culture(s) of learning, assessment, and practice; evaluation of impact; sustainability; and funding.

**Conclusions:**

Overall, this review describes a diverse group of programs that have been or are currently being implemented through partnerships. Additionally, it highlights several areas for program development, including addressing other topic areas within EM beyond trauma and ultrasound and evaluating outcomes beyond the level of the learner. These steps to develop effective programs will further the advancement of EM as a specialty and enhance the development of effective emergency care systems globally.

## Introduction

The World Health Assembly Resolutions 60.22, 68.15, and 72.16 all highlight the significant gap between the need for and the provision of emergency care that currently exists globally [1,2,3]. Low-income countries carry more than three times the burden of disease for emergency conditions in terms of disability-adjusted life years (DALYs) than do high-income countries, while emergency usage rates in low-income countries are 3% of those of high-income countries [4]. As more and more countries experience population and economic growth, globalization, rapid urbanization, and progression through the demographic transition, the demand for strong systems to address both the care and prevention of trauma, communicable diseases, and non-communicable diseases will only continue to grow.

Emergency care systems – which broadly include the provision of both acute and emergency care and services – are comprised of at least five major components: individual and community capacity, preparedness, and resilience; delivery of appropriate out-of-hospital care by laypersons; a means of access to formal services by the public; transport and/or delivery of appropriate pre-hospital care by trained professionals; and clinical care and referral networks [5,6]. The specialty of emergency medicine (EM) – a unique discipline with a well-defined and universal set of technical and cognitive skills – has developed over the last 50 years to lead the provision of clinical care by training not only physicians, but also other healthcare personnel at all levels. Additionally, the specialty of EM also serves as a guide for the overall development of emergency care systems through a focus on core concepts and strategies aimed at reducing morbidity and mortality of emergent conditions and to provide secondary disease prevention [5,6,7,8,9,10].

Although still a relatively young specialty, EM has been expanding rapidly across the globe, and more than 50 national EM organizations are now members of the International Federation for Emergency Medicine (IFEM) [11]. Although some concepts, such as burden of disease and resource availability, are unique to each local context, the specialty of EM provides a unique opportunity where partnerships can develop to share universal content as well as established resources in education and training relevant to high-resource settings, low-resource settings, or both. To date, the development of EM in many countries has occurred in part because of educational partnerships between nations or regions where one partner has more experience with EM as a specialty. Such partnerships can occur regardless of each country’s income level, but frequently involve partnerships between a high-income country and a low- or middle-income country (LMIC) [12,13,14,15].

Though programs developed from such educational partnerships are increasingly common, there is little to describe their features. One review describes some of the features of training programs in LMICs, but it does not specifically focus on programs resulting from partnerships [16]. Additionally, there is some guidance in the literature as to how to approach the creation of these training programs as well as the overall development of EM, but this information is now outdated and there is little information specific to training and education [17] In this systematic review, we aim to address this gap by highlighting the scope, content, and features of EM programs developed in LMICs through partnerships.

## Methods

An electronic search using PubMed, Web of Science, CINAHL, and EMBASE was conducted for all available publications to the date of the search (30 August 2018) without restriction on the scope of the EM content, the training level of the learners (i.e. medical student, nurse, mid-level, physician), or the length of the program (defined here as short duration – up to one month, medium duration – between one month and one year, and long duration – more than one year). The search strings may be found in Supplement 1. We have defined an EM training program as “structured education and/or training in the methods, procedures, and techniques of acute or emergency care”. Inclusion criteria determined *a priori* were EM training programs developed in the hospital or clinical setting in an LMIC – as defined by the World Bank at the time of the search – and supported by at least one higher-resource international partnership [18]. In-hospital programs were targeted as, in our experience, partners often seek out institutions given the often more familiar workforce and resources as well as the perception that training is likely to have the highest impact in this setting. Manuscripts focusing only on pre-hospital or non-clinical providers were excluded. Manuscripts with a predominant focus on other specialties (i.e. obstetrics/neonatal care and emergency surgery) were excluded. Only manuscripts in English were considered. Systematic reviews and abstracts/posters were excluded. Two reviewers screened each abstract with conflicts resolved after a third review to determine the included manuscripts.

The included manuscripts were then scored by the reviewers in order to select and more closely examine a high-quality subset. Each manuscript was scored by two reviewers and manuscripts with scores differing by more than three points (n = 13) or that differed between reviewers across the threshold for inclusion (n = 19) were reviewed and scored a third time. Scoring (0–2 points for each category) focused on seven areas: manuscript design, overall quality, overall clarity, ethical considerations, significance, description and/or study of program attributes and/or learner or clinical outcomes, and type of providers (Supplement 2). The methods used for this scoring were adapted from methods established in other reviews in the field of global EM [19]. The reviewers’ scores were then averaged for a final score on each manuscript. On review of the average scores, the upper quartile was found to be designated by a score of 12 and above. Manuscripts were then labeled as ‘low-scoring’ (below 12) or ‘high-scoring’ (12 or above).

Abstracts were screened using Covidence (Melbourne, Australia). Statistical analyses (Krippendorf’s alpha for nominal and for interval data for two or more reviewers) were done using Microsoft Excel and Stata/IC v15.1 (StataCorp; College Station, Texas USA). The study was registered and approved by PROSPERO (Registration: CRD42018100194).

## Results

**Figure 1:**
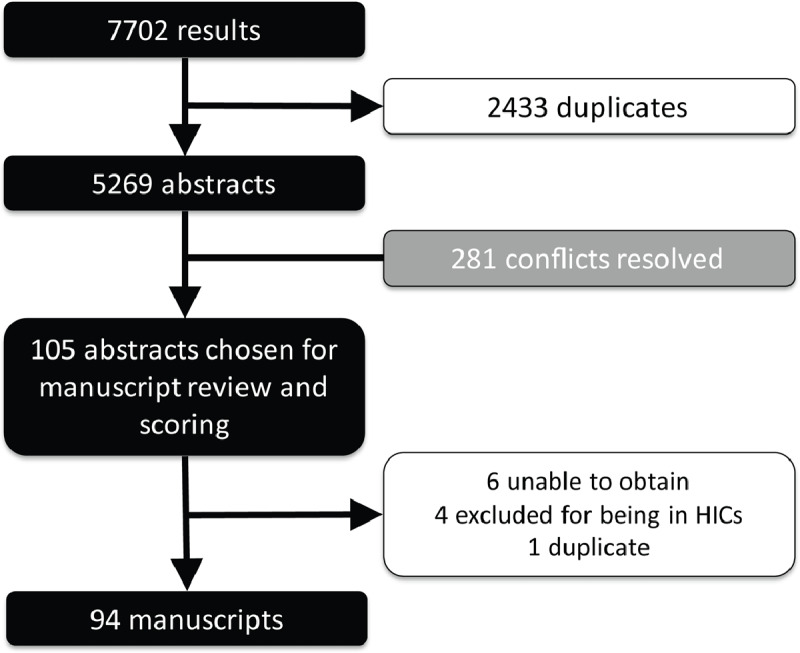
PRISM chart.

**Figure 2:**
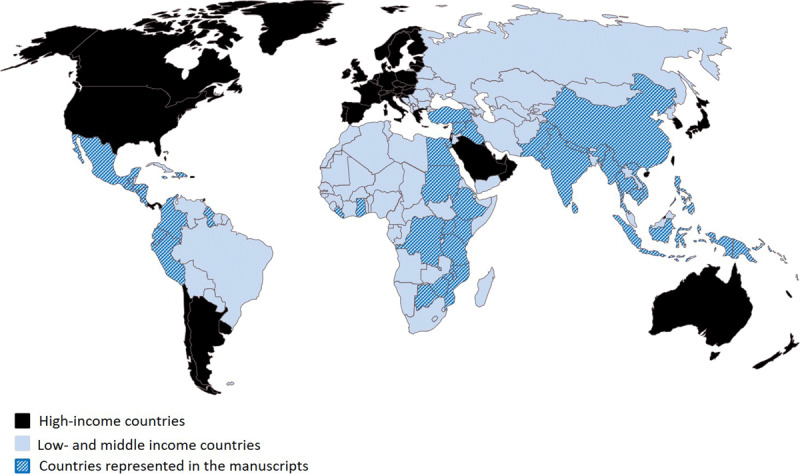
Low- and middle-income countries with programs highlighted in the 94 abstracts.

The search produced 7702 results. A total of 2433 duplicates were removed prior to screening, leaving 5269 studies to be screened by two reviewers. From this, 105 manuscripts met inclusion criteria for scoring. Of these, four were found to be in high income countries, six could not be obtained, and one was found to be a duplicate, leaving 94 manuscripts in the final analysis (Figure 1). Programs in 42 countries are represented in these manuscripts (Figure 2).

Of these manuscripts, 30 focus on EM programs with a broad scope, 18 focus on trauma training, 17 on pediatric training, and 16 on ultrasound training. Most programs focus on physician-learners; however, 17 programs train physicians and nurses together, and 15 train multidisciplinary teams (physicians and/or nurses along with other clinical and non-clinical professionals and/or staff). Additionally, eight programs focus solely on nurses and six on medical students. Finally, more than half of the programs are of short duration. Only three are of medium duration, and 15 are of long duration (Table 1).

**Table 1:** Summary of the 94 included manuscripts.

Characteristics	Number of Manuscripts

**Content**	

Broad EM	30
Trauma	18
Pediatrics	17
Ultrasound	16
Procedures	5
Pediatric Trauma	2
Disaster	2
Toxicology	1
Neurology	1
CPR	1
Maternal and Child Health	1
**Learners**	

Physicians	33
Physicians and Nurses	17
Multidisciplinary*	15
Nurses	8
Medical Students	6
Health Workers	4
Physician Assistants/Nurse Practitioners	1
Unspecified	10
**Program Duration**	

Short (up to 1 month)	51
Medium (>1 month to 1 year)	3
Long (>1 year)	15
Unspecified	25

* Physicians and/or nurses along with other clinical and non-clinical professions and/or staff.

The manuscripts were then scored in order to highlight a high-quality subset. When assessing the inter-rater reliability of the raw scores among the reviewers, the reviewers’ scores agreed +/–2 points 74.7% of the time (Krippendorff’s alpha 0.732). When assessing the inter-rater reliability to distinguish “low-scoring” from “high-scoring” manuscripts, the reviewers agreed 80.0% of the time (Krippendorff’s alpha 0.526). A total 26 manuscripts received an average score of 12 and above.

### Overview of High-Scoring Manuscripts

Fifteen of the high-scoring manuscripts focus on seven specific content areas (ultrasound, trauma, pediatrics, pediatric trauma, neurological emergencies, toxicological emergencies). They span the years 2012–2018, are published in twelve journals, and highlight work in thirteen countries (Table 2) [20,21,22,23,24,25,26,27,28,29,30,31,32,33,34]. Eleven of the manuscripts discuss general EM programs. These span the years 1999–2018, are published in 10 different journals, and highlight work in nine countries (Table 3) [35,36,37,38,39,40,41,42,43,44,45]. The components of the eleven manuscripts on broadly focused EM training programs are further highlighted here. Post hoc review of the other 83 included manuscripts demonstrated that results and the conclusions drawn from the 11 high-scoring manuscripts were representative of the larger group.

**Table 2:** Overview high-scoring manuscripts on content-specific EM programs.

First Author	Journal	Year Published	Low-resource Setting	Higher-resource Partner	Learners	General Overview (short: up to 1 month; medium: >1 month to 1 year; long: >1 year)

Becker	Trop Med Int Health	2017	Ghana	USA	Physicians	Ultrasound, unspecified duration
Cioè-Peña	Trauma	2017	El Salvador	USA	Physicians and Nurses	Trauma, short duration
Crouse	Pediatr Emerg Care	2016	Guatemala	USA	Physicians, Nurses, Medical Students, Others	Pediatrics, short duration
Denny	World J Emerg Med	2018	Tanzania	USA	Physicians, Students, Others	Ultrasound, short duration
Henwood	Trop Med Int Health	2016	Rwanda	USA, Canada	Physicians	Ultrasound, short duration
Kapoor	Emerg Med J	2016	Guatemala	USA	Physicians and Nurses	Pediatric Trauma, short duration
Kesinger	Injury	2014	Colombia	USA	Physicians	Trauma, unspecified duration
Merchant	World J Surg	2015	Mozambique	USA	Hospital Personnel, Others	Trauma, short duration
McCredie	Int Health	2018	Nepal	Canada, UK, USA	Physicians	Neurological Emergencies, short duration
Meaney	Resuscitation	2012	Botswana	USA	All Healthcare Providers	CPR, short duration
Reynolds	AfJEM	2016	Tanzania, Mexico	USA	Physicians	Ultrasound, short duration
Senarathna	PLoS One	2013	Sri Lanka	Australia	Hospital Staff	Toxicological Emergencies, short duration
Stolz	Trop Med Int Health	2015	Uganda	USA	Nurses	Ultrasound, long duration
Tafoya	J Emerg Med	2017	Ghana	USA	Physicians	Ultrasound, short duration
Wanjiku	AfJEM	2017	Kenya	USA	Medical Students	Trauma, short duration

**Table 3:** Overview high-scoring manuscripts on broad EM programs.

First Author	Journal	Year Published	Low-resource Setting	Higher-resource Partner	Learners	General Overview (short: up to 1 month; medium: >1 month to 1 year; long: >1 year)

Aggarwal	J Emerg Trauma Shock	2014	India	USA	Physicians	Comprehensive long residency program
Hammerstedt	Ann Emerg Med	2014	Uganda	USA	Nurses	Comprehensive long course; development of new provider level
Keyes	Ann Emerg Med	1999	Costa Rica	USA	Physicians	Comprehensive long residency program
Lim	Afr J Health Professions Educ	2017	Tanzania	USA, South Africa	Physicians	Comprehensive long residency program
Meshkat	BMC Med Educ	2018	Ethiopia	Canada	Physicians	Comprehensive long residency program
Mahadevan	AEM Educ Train	2018	Uganda	USA	Medical Students	Online vs classroom-based training; short course
Niyogi	AfJEM	2015	Ghana	USA	Physician Assistants	Task-shifting; short course
Pean	Ann Global Health	2015	Haiti	USA	Medical Students	Bilateral exchange; short course
Reynolds	J Public Health Policy	2012	Tanzania	USA, Canada, South Africa	Physicians and Nurses	Comprehensive training at multiple levels up to residency program level (medium and long)
Rouhani	Int J Emerg Med	2018	Haiti	USA	Physicians	Bridging the gap to residency programs; comprehensive medium-duration course
Stanley	Confl Health	2015	Thailand/Myanmar	UK	Nurses (and medics)	Primary care setting/front line providers; short course

### Broadly-focused EM Training Programs: Program Components

Participants include nurses (two programs), medical students (two programs), physician assistants (one program), physicians (five programs), and nurses and physicians together (one program). All of the manuscripts describe all or a subset of the following components: goals/objectives, program certification/recognition, curriculum outline/content, methods of assessment, funding, logistics, educational resources, outcomes, and challenges (Table 4).

**Table 4:** Program components from the selected manuscripts on broad EM programs.

First Author	Goals and Objectives	Certification/Recognition	Curriculum Outline/Content	Methods of Assessment	Funding	Logistics	Educational Resources	Outcomes	Challenges

Aggarwal	To provide guidelines for EM curricula in India – residency and medical student	Formal residency training	Core topics and required procedures for residents outlined; off-service rotations (minimum two weeks); medical student rotation (one month); no specific academic activities for medical students; required Casualty (two weeks) or EM (four weeks) posting during internship; no specific academic activities for interns; academic activities are department-wide	Thesis requirement; end-of-rotation assessment; log book; final examination with theory and practical components with short-answer questions, short cases, procedural skills (simulated), and OSCEs	Not discussed	Three-year training program; institutional requirements for ED resources and capacity and EM instructors defined	Calls for re-organization/re-structuring of existing resources; lists commonly used books and journals; reports essential list for departmental library	Clear requirements for institutions for the practice of and training in EM	Ongoing conflict and confusion with other specialty degrees such as trauma and surgery and critical care
Hammerstedt	To deliver emergency care through non-physician providers (Emergency Care Practitioners, or ECPs) in rural, low-resource settings	Emergency Care Practitioner certification	Graduated clinical and educational responsibilities; research and quality improvement requirements; weekly conferences comprised of didactic lectures, simulation, and procedural skill laboratories First year (Junior ECP) – 40 hours per week in beside learning; three hours of conference per week Second year (Senior ECP) – lead the junior-level conferences; present morbidity and mortality lectures; bimonthly CME classes on topics such as teaching	Intermittent quizzes and final written and oral examinations; procedure and patient follow-up logs; regular feedback with program director; oral case-based remediation test if needed; regular evaluation by visiting emergency physicians	Non-profit organization (Global Emergency Care)	Two-year program, train-the-trainer model	Core competencies from Uganda’s Medical Education for Equitable Services to All Ugandans and the US’s Accreditation Council for Graduate Medical Education Outcome Project; content developed by global emergency care physicians, the hospital medical superintendent, and faculty from Mbarara University and the Ministry of Health	Emergency care practitioners trained; plans to monitor patient outcomes and to expand to other sites	Tracking outcomes; expansion to other sites; funding; continued international faculty support
Keyes	To develop EM as a specialty in Costa Rica	Certificate for the Faculty Preparation Course; formal residency training	Faculty Preparation Course: review of core EM topics and instruction in teaching techniques – two hours on case review and journal club and four hours of lectures as well as clinical rotations (200 hours) and workshops; two-thirds of lectures in flipped classroom format Residency: overlap with the Faculty Preparation Course as well as a preceptor program and weekly grand rounds	Written and oral examinations monthly	Project HOPE, People-to-people Health Foundation and USAID	Faculty Preparation Course on-site by international faculty (one year) followed by the residency program (three years) supported by the first faculty for at least five years; learners included foreign physicians	ACLS, ATLS, PALS; US residency materials adapted to the local setting	14 graduates from the residency as of 1999	Attrition; payment for/status of physicians after graduation
Lim	To determine the acceptability of small-group learning in EM training	Formal residency training	Initially a lecture-based format in 2010 and redesigned to include small-group learning (40% of the curriculum) in 2014 including case-based seminars, procedure labs, and resuscitation simulations	Described elsewhere – written, oral and practical examinations; quantitative and qualitative survey	Described elsewhere – Abbott Fund Tanzania along with a departmental business plan for financial sustainability	Small groups done weekly (seminars) or 1–2 times per month (procedure labs, simulations) with an instructor-learner ratio of 4-6:1	Described elsewhere – African Federation for Emergency Medicine; International Federation for Emergency Medicine	Small groups more effective at improving clinical practice and preferred for enjoyment of learning and peer- and instructor-relationship building; preferred by learners with more experience	Novel type of learning; some sessions remain too “lecture-like”; relationship building may be difficult across learner levels
Meshkat	To develop and deliver comprehensive EM residency training; specific objectives outlined in the manuscript	Formal residency training	Clinical, Clinical Epidemiology, and EM Administration streams; didactics (separated into blocks), beside teaching, simulation, procedural sessions, and journal club; three half days a week for three months of the year; off-service rotations; formal faculty-resident mentorship program; monthly video conferencing	Session and rotation evaluations; written and practical examinations	University-based funding; Grand Challenges Canada; International Development Research Centre; hospital-based funding	Three-year program with annually repeating junior lecture series and bi-annually repeating senior series; three one-month teaching trips by visiting faculty/senior residents; briefings for visiting faculty; curriculum co-director oversight	Core content based on facility assessment, learner needs assessment, and evaluation of disease burden; free and open-access EM-specific materials listed; University of Toronto postgraduate medical education documents used to develop evaluation methods	The program is in its seventh year as of 2018 with 34 graduates, 20 working in EDs throughout Ethiopia, and 25 modules published online	Development of content by outside experts leads to discordance with local practice and resources; the program was time and resource-intensive in first three years; gaps in visiting faculty led to low morale; internet connection with teleconferencing occasionally poor
Mahadevan	To determine differences in knowledge acquisition between online and classroom-based teaching on EM concepts	None	20 modules; 10–15-minute videos or in-person teaching; case-based	Multiple choice and free-response questions	University-based grant	The online course was offered during 10 weeks of the academic year and the classroom-based course was completed over one week during the school break	Novel course developed by Stanford physicians; clips from the show *ER*	Both groups improved their scores on the post-test with no significant difference overall between the two groups	High numbers of late enrollees and attrition, especially in online course; the online group post-test was delayed by three months
Niyogi	To increase knowledge and to allow for task-shifting in the delivery of emergency care by teaching physician assistants (PAs) to identify and stabilize patients with acute conditions; focus on ABCs	In-service training in the Ghana Health Service	Didactic lectures, problem- and case-based small group sessions, skill stations, simulations, laminated algorithms, and a tabletop mass casualty incident exercise; initial training of trainers and supervision of initial trainings by international faculty	Written testing; observation of learners; case review; simulations	Not described	Nine-day trainer training with a refresher six months later; five-day in-service trainings led by trainers in groups of 2–3	ABCCC approach from the Integrated Management of Adolescent and Adult Illnesses and the Integrated Management of Childhood Illnesses; Ghana Standard Treatment Guidelines	All post-test scores improved from pre-test scores; the trainer refresher pre-test scores fell nearly to initial pre-test levels, but regional pre-test refresher scores stayed relatively high; 22 initial senior trainers, 39 enrolled in initial regional courses	Decay in knowledge over time; minimal differences in knowledge between trainers and trainees; resource limitations at non-training sites; scope of practice limitations for PAs; lack of familiarity with leading/facilitating case-based methodologies and simulation; refresher courses delayed too long; inability for trainees to leave clinical requirements for the training
Pean	To increase knowledge and confidence in emergency response skills using a near-peer model	BLS certification	BLS (three days); EM Module – lectures and skills sessions (two days)	Written examination and observed practical skills examination; fund of knowledge tests and self-efficacy surveys; one-year follow up survey	Self-funded and/or sponsored by private donations as well as Doctor’s Hospital at Renaissance in Edinburg, Texas, and the Icahn School of Medicine at Mount Sinai in New York, New York	One week annually for two years; student to instructor ratio 3:1; follow up survey given during the second year; lectures in the morning followed by skills sessions in the afternoon	BLS resources supplied by the Regional Emergency Medical Services Council of New York City; STRAKER Translations for translating written material; EM Module from the introductory course at Icahn School of Medicine at Mount Sinai	Improvements in fund of knowledge and self-efficacy test scores	Unexpected scheduling changes; language barrier; difficulty with maintaining continuity and connection among cohorts; high levels of non-completion and absenteeism; difficulties in communicating expectations; limited access to electricity
Reynolds	To provide multiple levels of training to provide emergency care	Formal residency training – Master of Medicine track	International faculty with transition to local faculty; competency-based; half of the time is spent on rotations in other specialties	Written multiple choice questions and essay exam; oral case-based exam; observed clinical exam (OSCE) with case presentation; professional performance audits	Abbott Fund Tanzania along with a departmental business plan for financial sustainability	10-module nursing curriculum; 1-year registrar program; 3-year residency program	African Federation for Emergency Medicine; International Federation for Emergency Medicine	90% of nurses have completed the program; residency graduates since 2013; credentialed registrars	Differences in scope of practice in low-resource settings – curricula and off-service rotations must be modified accordingly; culture of practice slow to change
Rouhani	To address a gap in human resources and knowledge in EM until more physicians are able to complete formal residency programs	Formal certificate with the Ministry of Health and National Medical School	Didactic lectures, simulation, journal club, morbidity and mortality conference, skill sessions clinical supervision, and development and delivery of one lecture; learners comprised of physicians from multiple hospitals; international and national faculty	Attendance of 75%, 180 supervised clinical hours, written pre- and post-test, completed case and procedure log	Course free of charge; participants retained full salary; non-local participants provided food and housing; subsidized in-country expenses for visiting faculty	6-month course; 96-hour didactic program conducted every other week; eight visiting faculty volunteering 3–4 weeks	Use of established residency resources; ACLS and PALS resources	11/14 graduates still working in Emergency Departments one year later; average improvement of 15 points between pre- and post-test scores	Frequent turn-over of clinical supervisors; resource limitations at non-training sites; views of staff/culture of institution; lack of curriculum flexibility; varied baseline knowledge; language barrier
Stanley	To improve competence in assessment and management of emergent conditions	None	Scenario-based drills/training with whiteboard and reference cards; focus on ABCDE	Observed scenarios (OSCE) and feedback form; pre-assessment; 1–2-week post-assessment and 8-week assessment	Not described	Three sites; four days at each site (pre-assessment, training, post-assessment, 8-week assessment) with two identical sessions daily	Previously used local training tools and instructor experience	Assessment scores and self-reported confidence scores all improved after the intervention	Attendance low

### Goals and Objectives

Each of the eleven programs conducted formal needs assessments and/or had focus group discussions to identify learning gaps and to determine the content, style, and/or duration of each program, fulfilling the first two steps Kern et al.’s six-step model for curriculum development [46]. These efforts drew upon the strengths and experiences of the instructors while considering the knowledge level of the learners and using familiar teaching styles and/or providing orientation to new instructional methods. One manuscript highlights a pilot study prior to full implementation of a program [35]. Three studies highlight instructor (both international and local) development and orientation prior to commencement of the program [38,41,42].

### Certification/Recognition

More than half of the manuscripts discuss alignment with local or national governing bodies, such as the Ministry of Health (MoH) regarding formal recognition, licensing, and/or certification of their respective programs.

### Curriculum Outline/Components

The curricula highlighted in these manuscripts span short, foundational programs to medium-length programs with abbreviated, comprehensive EM curricula to full-length residency programs (of at least three years). All offer mixed learning methods ranging from traditional didactic lectures and small group discussions (i.e. journal clubs and case-based sessions) to hands-on practical sessions and workshops, such as simulation and procedural training. Curricula are often organized into blocks or modules by topics or organ systems. Longer programs allocate one to three half-days every one or two weeks for learning sessions in addition to daily bedside clinical teaching. Volunteer patients, mannequins, laminated algorithms, white boards, and other simulation materials as well as video/online materials, handbooks, and textbooks are highlighted as curriculum tools. Many programs incorporate pediatrics as well. All residency programs list the requirement of off-service rotations, such as surgery, anesthesia, and critical care. Only one manuscript offers extensive online access to their curriculum map and resources [41]. One of the short programs emphasizes the need for refresher courses, particularly when limited curricula are offered and/or baseline knowledge is varied or limited [39]. One manuscript specifically evaluates the acceptability of a novel-to-context learning modality (small group sessions) within the overall program [45].

### Methods of Assessment

All programs have a written component of assessment. These examinations consist of multiple-choice questions, short-answer, and/or essay questions. Many manuscripts highlight programs that also have practical examinations – skills stations, oral and/or case-based examinations as well as objective structured clinical examinations (OSCEs). Longer programs also often track attendance and require case and/or procedure logs with minimum required numbers. Minimum passing requirements for examinations and attendance, when specified, range from 70–80%. There are often end-of rotation evaluations and performance audits, especially for residency programs with off-service rotations. There is usually ongoing informal feedback and mentorship. One program mentions requiring a thesis [44].

### Funding

Three of the 11 manuscripts do not discuss how their programs are supported, while the others note a combination of private donors, non-profit organizations, healthcare organizations, and funding from universities in higher-resource settings. Manuscripts that mention their funding sources note a mix of self-funding or subsidized funding for international faculty. Among the larger funding sources listed are the Abbott Fund, USAID, Grand Challenges, and Project HOPE. Salary for learners in residency programs and fees for learners for non-residency programs are less clear – at least one program notes that there are no fees for their learners, and those who had to travel for the program received room and board [36].

### Logistics

All of the programs involve international visiting faculty for ongoing full or supervisory support either in person and/or via teleconferencing, messaging, or other methods of remote engagement. Visiting faculty contributions are described as ranging from one week up to multiple years onsite and/or remotely. Longer programs tend to have curricula that repeat one or more times over the course of the program. Train-the-trainer techniques are used in two of the programs [39,43]. Two programs mention their learner-to-instructor ratio for hands-on sessions (a range of 3–6:1) [38,45]. One program is structured to have two identical sessions daily in order to accommodate the learners, who must provide continuous coverage in the clinical setting [35].

### Educational Resources

Content for the curricula is drawn from a variety of sources – usually initially from EM content belonging to the high-resource partner. Specific materials and organizations listed among the programs for reference include basic life support (BLS), advanced cardiac life support (ACLS), advanced trauma life support (ATLS), pediatric advanced life support (PALS), textbooks (specifically Tintinalli, Rosen, and Goldfrank, among others), World Health Organization (WHO) resources (such as Integrated Management of Adolescent and Adult Illness and Integrated Management of Childhood Illnesses), Canadian Association of Emergency Physicians (CAEP), American College of Emergency Physicians (ACEP), IFEM, Society for Academic Emergency Medicine (SAEM), African Federation for Emergency Medicine (AFEM), European Society for Emergency Medicine (EuSEM), World Association for Disaster and Emergency Medicine (WADEM), Australasian Society for Emergency Medicine (ASEM), Consortium of Universities for Global Health (CUGH), Johns Hopkins Global Health, international EM journals (i.e. *International Journal of Emergency Medicine*), and American and Canadian graduate medical education (GME) resources.

### Outcomes

Knowledge acquisition, clinical performance, and/or learner confidence/self-assessment are among the outcomes listed for almost all of the programs. All programs conduct immediate assessments; however, many of the programs also assess knowledge retention at a variety of time points: eight weeks, three months, six months, and one year. Additionally, some programs highlight the number of graduates as well as the number of individuals still practicing in EM. None of the program outcomes extend beyond the learner, although future studies of clinical outcomes are mentioned in one manuscript [43].

### Challenges

Challenges common to many of the programs include establishing the status and culture of practice of EM in low-resource, novel settings. Additionally, the manuscripts describe how differences in scope, practice, and content between the high-resource and low-resource settings as well as among program sites within low-resource settings require translation and adaptation of any shared educational resources and techniques. Overall resource limitations for learning as well as practice materials are frequently noted. Language barriers (for both instruction and materials) as well as issues with remote connection and use of local and remote technologies are also highlighted. Additionally, lack of continuity and connection between learners and the rotating visiting faculty – who may be present in person for as little as one week – leads to reduced engagement and morale. Decay in knowledge, especially after short programs, is also a concern, with re-assessment between 2–3 months highlighted as potentially an ideal time for re-assessment or refresher training. Difficulties with both learner (e.g. attendance given the requirement of ongoing continuous clinical duties still required during many of the programs) and curriculum flexibility leads to attrition for many programs. There are also ongoing concerns regarding sustainable funding highlighted in several of the manuscripts – for the program, for visiting faculty, as well as for learners/graduates.

## Discussion

These manuscripts describe many types of EM programs being implemented via partnerships worldwide to train in-hospital personnel critical for the overall development of emergency care systems – from short, focused courses on topics such as ultrasound training or trauma training to full residency programs. The most established programs are those that are of at least one year in duration and those with formal integration into the healthcare and/or medical education system of the country (e.g. supported by the MoH). These programs can then offer official certification and/or credentialing to ensure that graduates will have both potential positions and financial security as well as a career path as EM develops and the culture of practice changes.

All of the programs involve on-site and/or remote international faculty for varying lengths of time; however, programs in which at least a core subset of faculty are involved in face-to-face teaching through the duration of the program foster stronger partnerships and allow for continuity of evaluation as well as continuous improvement and adaptation of the program. Given the differences in resources and culture of practice, all visiting faculty need at least prior experience in education/training and low-resource environments and/or a comprehensive orientation to the site prior to providing instruction. One manuscript describes the challenges of conflicting expectations between instructors and learners due to the limited preparation and experience by the visiting faculty [38].

Furthermore, the use of international faculty is rarely sustainable, especially when support for these faculty is not available. Programs that involve a train-the-trainer component as well as faculty development are essential, although limitations of these models (especially dilution of instruction) must be kept in mind and addressed using other mechanisms such as refresher courses and mentorship or preceptor programs. Additionally, given the increasing familiarity with and use of technology, non-traditional training mechanisms such as the use of messaging applications and other mobile technology-based training tools should be considered, especially as they can be effective mechanisms to extend training beyond face-to-face interactions and/or to continue training during protracted and/or complex emergencies [47,48,49].

These programs also highlight the inability to simply transfer EM curricula from one setting to another. Some fundamental concepts can be shared, as is evidenced by international guidelines for curricula, and there are several groups that have offered open-access materials that are already adapted to low-resource settings to varying degrees (Table 5). In order to be effective, however, the development of curricula – whether abbreviated or a full residency program – must be preceded by needs assessments, focus-group discussions, and knowledge of the epidemiology and burden of disease as well as the culture of practice, instruction, learning, and assessment of the implementation site [50,51,52]. Additionally, language barriers must be overcome both in instruction as well as in written and electronic materials [53]. Finally, there must be inter-sectoral engagement and collaboration between the health and education sectors in order to recruit, develop, and sustain a quality workforce that is matched to overall service delivery capacity and population needs [54].

**Table 5:** Sample list of curricula and content resources.

Organization	Resources	Website*

Global Health Emergency Medicine (GHEM)	Teaching modules	http://ghem.ca/modules/
African Federation for Emergency Medicine (AFEM)	Curricula, lecture bank, ultrasound lecture bank	https://afem.africa/resources/
World Health Organization (WHO)	Full short course	https://www.who.int/emergencycare/publications/Basic-Emergency-Care/en/
International Student Association of Emergency Medicine (ISAEM) and the International Emergency Medicine Education Project (iEM)	Medical student resources	https://isaem.net/ https://iem-student.org
International Federation for Emergency Medicine	General guidelines; curricula outlines	https://www.ifem.cc/
OPENPediatrics	Pediatric-specific resources	https://www.openpediatrics.org/
Emergency Ultrasound Teaching	Ultrasound-specific resources	http://emergencyultrasoundteaching.com/
Free, Open-Access Medical Education Resources	One list of general resources	https://rebelem.com/focused-foamed-the-learners-lens/
American College of Emergency Physicians (ACEP) International Section	General resources from an EM organization	https://www.acep.org/how-we-serve/sections/international-emergency-medicine/free-educational-resources/

* Accessed: May 2019; the authors do not specifically endorse any particular resource, nor do they verify the accuracy of the content listed at the sites above.

Finally, in order to continue to advance EM training and the overall provision of emergency care, programs must include training in quality improvement (QI) and research, topics not traditionally taught in many medical and nursing schools in low-resource settings. To illustrate this point, less than half of the programs mention a component of QI and/or research, and only two of the manuscripts have a local first author [31,44]. Furthermore, in order to determine which methods of training are most effective, we must move beyond metrics regarding knowledge acquisition and attitude and eventually look to patient-, community-, and population-based outcomes [55]. Only three of the manuscripts evaluate patient and/or clinical outcomes as a result of their training programs [20,26,31].

Despite ongoing work to develop metrics and quality indicators for emergency care and research and to provide guidance in the development of emergency care systems (as evidenced by the WHO’s Emergency, Trauma and Acute Care Program), there is still very little research that has focused specifically on the impact of training programs [9,56,57,58,59,60,61,62,63]. These programs are an integral part of the foundation of a sustainable emergency care system, not only at the facility level, but indirectly within the community and the pre-hospital system as well, therefore, further investigation of and research on these programs is imperative to sustain and to continue to expand EM as a specialty and emergency care systems globally.

## Limitations

Overall, we used only four search engines and excluded programs in the gray literature without peer review (i.e. abstracts) – an area in which many more programs are likely highlighted based on an informal review of the excluded abstracts. Also, our results are limited to manuscripts only in English. Additionally, the search terms used to define this rapidly changing and developing field were difficult to specify, leading to one additional manuscript that was identified on informal review that could have been included in this study [64]. Furthermore, partnerships are also not always made explicit in many manuscripts; since our study focused on programs developed through educational partnerships, we may have missed manuscripts in the screening process where this was not clearly defined [65]. This challenge further emphasizes the need for standardization of practice and sharing of knowledge and experience in this field. Finally, our scoring process was developed from tools used in other reviews and was not formally tested with our results; however, it has been used to evaluate literature in field of global EM, and post hoc review demonstrated that the conclusions drawn from the high-scoring manuscripts were representative of the larger group [19]. Despite these limitations, however, this study still identifies a broad range of EM training programs, and it highlights programmatic components essential for effective partnerships and successful implementation.

## Conclusions

Training in EM is an essential component to developing strong emergency care systems. This study identifies a broad range of EM training programs developed through partnerships that are representative of the programs that exist globally and highlight programmatic components essential to the development of effective programs. Sharing knowledge and practice across high- and low-resource settings can be an effective mechanism for the global development of programs within a specialty, however significant adaptation to the educational setting as well as true investment and partnership must occur, even for short-term programs. Even when all of the complexities and components are addressed, there are still limitations to this practice. Short-term programs need to expand beyond the few topics that are represented now, and we must invest in comprehensive, long-term emergency care training and professional development programs. Finally, as tools and guidance regarding the development of emergency care systems increase, a strong focus on strengthening educational practices as well as evaluating their impact through research is imperative.

## Additional Files

The additional files for this article can be found as follows:

10.5334/aogh.2681.s1 Supplement 1. Search Strings.

10.5334/aogh.2681.s2 Supplement 2. Scoring Rubric.
