# Supplementary material for: Emergency Medicine Training Programs in Low- and Middle-Income Countries: A Systematic Review
Source: Ann Glob Health. 2020 Jun 16;86(1):60. doi: 10.5334/aogh.2681 (PMC7304456; doi:10.5334/aogh.2681)
Supplement: Supplement 1. — Search Strings. [file agh-86-1-2681-s1.pdf]

<u>Supplement 1: Search Strings</u>

PubMed:

(("Emergency Responders"[MeSH] OR "Emergency Medicine"[MeSH] OR "Emergency Medical Technicians"[MeSH] OR "Emergency Medical Services"[MeSH] OR "Pediatric Emergency Medicine"[MeSH] OR "Emergency Service, Hospital"[MeSH] OR "Emergency Services, Psychiatric"[MeSH] OR "Emergency Treatment"[MeSH] OR "Evidence-Based Emergency Medicine"[MeSH] OR Emergency health[tiab] OR emergency medicine[tiab] OR EM training[tiab] OR emergency care[tiab] OR emergency provider*[tiab] OR emergency service[tiab] OR (emergenc* AND medic*)) AND (learn* or instruct* or train* or educat* or course* or workshop* or work-shop* or program* or teach* OR curriculum* OR certificat* OR credential* OR medical education OR "Education"[MeSH] OR "education" [Subheading] OR "Learning"[MeSH] OR "Certification"[MeSH] OR "Credentialing"[MeSH] OR "Education, Medical"[MeSH] OR "Education, Medical, Continuing"[MeSH] OR "Teaching"[MeSH] OR "Curriculum"[MeSH]))) AND ("Developing Countries"[MeSH] OR "Poverty"[MeSH] OR "Poverty Areas"[MeSH] OR developing country[tiab] OR developing countries[tiab] OR developing nation[tiab] OR developing nations[tiab] OR less-developed countr*[tiab] OR less-developed nation*[tiab] OR third-world nation*[tiab] OR third-world[tiab] OR under-developed nation[tiab] OR underdeveloped nation[tiab] OR Resource-limited[tiab] OR middle-income countr*[tiab] OR middle-income nation*[tiab] OR low-income countr*[tiab] OR low-income nation*[tiab] OR poor countr*[tiab] OR poor nation*[tiab] OR lmic[tiab] OR lmics[tiab] OR LAMICs[tiab] OR LAMIC[tiab])

Web of Science:

(Indexes=SCI-EXPANDED, SSCI, A&HCI, CPCI-S, CPCI-SSH, BKCI-S, BKCI-SSH, ESCI, CCR-EXPANDED, IC Timespan=All years)

("Developing Countries"OR "Poverty" OR "Poverty Areas" OR "developing country" OR "developing countries" OR "developing nation" OR "developing nations" OR "less-developed countr*" OR "less-developed nation*" OR "third-world nation*" OR "third-world" OR "under-developed nation*" OR "underdeveloped nation" OR "Resource-limited" OR "middle-income countr*" OR "middle-income nation*" OR "low-income countr*" OR "low-income nation*" OR "poor countr*" OR "poor nation*" OR lmic OR lmics OR LAMICs OR LAMIC)

AND

(learn* OR instruct* OR train* OR educat* OR course* OR workshop* OR work-shop* OR program* OR programme* OR teach* OR curriculum* OR certificat* OR credential* OR "medical education" OR "continuing education")

AND

("Emergency Responders" OR "Emergency Medicine" OR "Emergency Medical Technicians" OR "Emergency Medical Service*" OR (Pediatric AND Emergency) OR "Emergency Service" OR (Emergency AND Psychiatric) OR "Emergency Treatment" OR "Evidence-Based Emergency Medicine" OR "Emergency health" OR "emergency medicine" OR (emergency AND medicine) OR "EM training" OR "emergency care" OR "emergency provider*" OR "emergency service" OR (emergenc* AND medic*))

CINAHL:

("Emergency Responders" OR "Emergency Medicine" OR "Emergency Medical Technicians" OR "Emergency Medical Service*" OR (Pediatric AND Emergency) OR "Emergency Service” OR (Emergency AND Psychiatric) OR "Emergency Treatment" OR "Evidence-Based Emergency Medicine" OR “Emergency health” OR “emergency medicine” OR (emergency AND medicine) OR “EM training” OR “emergency care” OR “emergency provider*” OR “emergency service” OR (emergenc* AND medic*)) AND (learn* OR instruct* OR train* OR educat* OR course* OR workshop* OR work-shop* OR program* OR programme* OR teach* OR curriculum* OR certificat* OR credential* OR “medical education” OR “continuing education”) AND ("Developing Countries"OR "Poverty” OR "Poverty Areas” OR “developing country” OR “developing countries” OR “developing nation” OR “developing nations” OR “less-developed countr*” OR “less-developed nation*” OR “third-world nation*” OR “third-world” OR “under-developed nation*” OR “underdeveloped nation” OR “Resource-limited” OR “middle-income countr*” OR “middle-income nation*” OR “low-income countr*” OR “low-income nation*” OR “poor countr*” OR “poor nation*” OR lmic OR lmics OR LAMICs OR LAMIC)

EMBASE:

'poverty' OR 'poverty areas' OR 'developing country' OR 'developing countries' OR 'developing nation' OR 'developing nations' OR 'less-developed countr*' OR 'less-developed nation*' OR 'third-world nation*' OR 'third-world' OR 'under-developed nation*' OR 'underdeveloped nation' OR 'resource-limited' OR 'middle-income countr*' OR 'middle-income nation*' OR 'low-income countr*' OR 'low-income nation*' OR 'poor countr*' OR 'poor nation*' OR lmic OR lmics OR lamics OR lamic

AND

learn* OR instruct* OR train* OR educat* OR course* OR workshop* OR 'work shop*' OR program* OR programme* OR teach* OR curriculum* OR certificat* OR credential* OR 'medical education' OR 'continuing education'

AND

'emergency responders'/exp OR 'emergency responders' OR 'emergency medical technicians'/exp OR 'emergency medical technicians' OR 'emergency medical service*' OR (('pediatric'/exp OR pediatric) AND ('emergency'/exp OR emergency)) OR (('emergency'/exp OR emergency) AND psychiatric) OR 'emergency treatment'/exp OR 'emergency treatment' OR 'evidence-based emergency medicine'/exp OR 'evidence-based emergency medicine' OR 'emergency health' OR 'emergency medicine'/exp OR 'emergency medicine' OR (('emergency'/exp OR emergency) AND ('medicine'/exp OR medicine)) OR 'em training' OR 'emergency care'/exp OR 'emergency care' OR 'emergency provider*' OR 'emergency service'/exp OR 'emergency service' OR (emergenc* AND medic*)
