# Supplementary material for: Emergency Medicine Training Programs in Low- and Middle-Income Countries: A Systematic Review
Source: Ann Glob Health. 2020 Jun 16;86(1):60. doi: 10.5334/aogh.2681 (PMC7304456; doi:10.5334/aogh.2681)
Supplement: Supplement 2. — Scoring Rubric. [file agh-86-1-2681-s2.pdf]

Supplement 2: Scoring Rubric

	Two Points	One Point	Zero Points
Manuscript Design	Formal assessment or evaluation of the outcomes of a program	If a report or commentary on a program	All other manuscript types
Ethical Considerations	If conflicts of interest (COI) declared AND (if applicable) IRB approval obtained AND adheres to the Declaration of Helsinki AND biases and/or limitations are described	If at least one, but not all applicable aforementioned conditions are met	If none of the aforementioned conditions are met
Quality	Proper analysis/statistics and tables/graphs used; appropriate sections (introduction/background, methods, results/conclusions) present	Minor errors in analysis/statistics; poor use of tables/graphs; some major sections incomplete	Major errors in analysis statistics; incomprehensible tables/graphs; major sections missing
Clarity	Overall clear and well-written	Lacks clarity overall	Unclear, poorly written
Significance	Significantly adds to overall understanding of implementation and/or impact of EM programs	Somewhat adds to overall understanding of implementation and/or impact of EM programs	Does not add to overall understanding of implementation and/or impact of EM programs
Program Attributes*	If five or more described	If two to four described	If none or one are described
Providers	Focused on EM-specific or general providers	Focused on providers in other specialties (i.e. Anesthesia, Surgery, Pediatrics, OB/GYN) OR a mix of EM and other providers	Type of providers not specified

*Program Attributes:
1. Goals and Objectives – clearly defined overarching goals with specific learning objectives outlined
2. Certification/Recognition – discussion as to process of formal certification/recognition/credentialing within the current healthcare system/structure
3. Curriculum Outline – details in terms of block structure, teaching/learning methods, topics covered
4. Methods of Assessment – examinations, clinical evaluation/feedback structure
5. Funding – sources of funding, program fees
6. Logistics – program administration, partnership logistics
7. Resources – resources drawn on for program development; open-source resources for other programs
8. Outcomes – participants (e.g. scores, knowledge retention) OR patients (e.g. clinical outcomes/metrics)
